# An association analysis to identify genetic variants linked to asthma and rhino-conjunctivitis in a cohort of Sicilian children

**DOI:** 10.1186/s13052-019-0603-4

**Published:** 2019-01-15

**Authors:** Gianluca Sottile, Giuliana Ferrante, Marta Torregrossa, Fabio Cibella, Giovanna Cilluffo, Salvatore Fasola, Riccardo Alessandro, Gregorio Seidita, Giovanni Viegi, Stefania La Grutta

**Affiliations:** 1 Department of Economics, Business and Statistical Science, University of Palermo, Viale delle Scienze, Building 13, 90128 Palermo, Italy; 2 Dipartimento di Promozione della Salute, Materno-Infantile, di Medicina Interna e Specialistica di Eccellenza “G. D’Alessandro”, University of Palermo, Palermo, Italy; 3 National Research Council of Italy, Institute of Biomedicine and Molecular Immunology, Palermo, Italy; 4 Dipartimento di Biomedicina, Neuroscienze e Diagnostica avanzata, University of Palermo, Palermo, Italy; 5 National Research Council of Italy, Institute of Clinical Physiology, Pisa, Italy

**Keywords:** Asthma, Rhino-conjunctivitis, Sicilian children, Genetics, SNPs

## Abstract

Asthma and rhino-conjunctivitis are common chronic diseases in childhood. In this cross-sectional study, we performed a gene association analysis with current asthma and rhino-conjunctivitis in a cohort of Sicilian children aged 10–15 years. Overall, our findings reveal the importance of different genetic variants at 4p14, 16p12.1, 17q12, 6p12.2 and 17q21.1, identifying possible candidate genes responsible for susceptibility to asthma and rhino-conjunctivitis.

To the Editor,

Asthma and rhino-conjunctivitis (RC) are common diseases worldwide that are frequently associated. Observed differences in prevalence of asthma and RC may be explained by genetic susceptibility, though environmental factors play a relevant role [1]. In order to increase genomic information on Sicilian children, this research has explored some genetic variants to discover possible association with asthma and RC.

A representative sample of 1050 children within the “Palermo Junior High School” (PJHS II) [2] study were investigated through questionnaires, spirometry, and skin prick test (SPT) to quantify the prevalence of asthma and RC, in association with allergic sensitization and respiratory function, and to evaluate the role of environmental and host risk factors for allergic respiratory diseases. The study was approved by the local Institutional Ethical Committee. All parents of the enrolled children signed a written informed consent.

Two different phenotypes were identified: Current Asthma (CA) defined as asthma ever plus at least a wheeze episode in the last 12 months, RC defined as sneezing, or runny, or blocked nose apart from common cold or flu in the last 12 months and nose problem accompanied by itching and/or watering eyes. The concomitant presence of CA and RC was merged into the CA group; children without CA and RC (nAnRC) were used as controls.

A total of 52 Single Nucleotide Polymorphisms (SNPs), involved in the innate immune system pathways were selected for genotyping by Matrix-Assisted Laser Desorption/Ionization (MALDI-TOF-MS). Out of the 52 initially selected SNPs, 7 were complete drop-outs and the remaining SNPs were successfully genotyped. The individuals were genotyped with the Illumina Bead-Chip (Illumina Inc., San Diego, CA, USA); the PLINK v1.07 software was used to perform standard quality control. SNPs were excluded if they had low call rates (proportion of genotyped called < 90%), were not in Hardy-Weinberg equilibrium (HWE, *p* < 0.001) on the nAnRC subjects, or had a low minor allele frequency (MAF < 1%). A total of 22 SNPs were used for further analyses.

Mean values were compared among children with CA, RC and nAnRC using the analysis of variance (ANOVA). Differences of categorical variables were evaluated using Chi-squared test. Associations between single SNPs and CA and RC were analysed by applying the case/control model of the SNPassoc R package, adjusting for sex, age, body mass index (BMI), SPT+ (at least one positive), exposure to current environmental tobacco smoke and traffic.

The demographic and lung function characteristics of the 1050 subjects are shown in Table 1. The study sample was composed by 523 (49.8%) Female and 527 (50.2%) Male, aged 12.07 ± 0.74 years on average. Subjects were categorized into CA (*n* = 61), RC (*n* = 184) and nAnRC (*n* = 805). Subjects with CA and RC more frequently had SPT+; subjects with CA were younger than nAnRC and RC subjects.

**Table 1:** Baseline demographic and clinical characteristics of study population

	nAnRC *n* = 805 (76.7%)	CA *n* = 61 (5.8%)	RC *n* = 184 (17.5%)	*p*-value
Female, n (%)	390 (48.45)	29 (47.54)	104 (56.52)	0.133
Age, mean (SD)	12.06 (0.74)	11.90 (0.62)	12.18 (0.74)	**0.025**
Height, mean (SD)	152.58 (7.87)	151.52 (7.81)	153.51 (7.41)	0.168
Weight, mean (SD)	49.05 (12.07)	50.11 (12.65)	50.28 (13.52)	0.417
BMI (kg/m^2^), mean (SD)	20.92 (4.28)	21.62 (4.15)	21.16 (4.78)	0.426
Skin Prick Test +, n (%) (#)	274 (34.16)	43 (70.49)	76 (41.30)	**< 0.001**
Environmental exposure *current*				
Tobacco smoke, n (%)	440 (54.93)	27 (44.26)	107 (58.15)	0.167
Traffic in the zone of residence, n (%)	627 (77.99)	41 (67.21)	134 (72.83)	0.071
Mould/dampness, n (%)	108 (13.47)	7 (11.67)	31 (17.03)	0.396
Spirometric values*				
FEV_1_%predicted, mean (SD)	100.39 (11.80)	96.96 (12.68)	98.82 (11.34)	**0.035**
FEV_1_ Z, mean (SD)	0.04 (1.02)	−0.26 (1.09)	−0.09 (0.98)	**0.037**
FVC %predicted, mean (SD)	97.10 (13.29)	97.60 (13.63)	95.51 (12.44)	0.304
FVC Z, mean (SD)	−0.27 (1.14)	−0.22 (1.17)	− 0.40 (1.07)	0.314
FEV_1_/FVC % predicted, mean (SD)	103.28 (7.57)	99.17 (8.11)	103.33 (7.89)	**< 0.001**
FEV_1_/FVC Z, mean (SD)	0.60 (1.21)	−0.04 (1.20)	0.60 (1.25)	**< 0.001**
FEF_25–75_%predicted, mean (SD)	102.10 (22.19)	89.17 (21.67)	101.76 (23.98)	**< 0.001**
FEF_25–75_ Z, mean (SD)	0.05 (0.98)	−0.55 (1.02)	0.02 (1.05)	**< 0.001**

*CA* current asthma, *RC* rhino-conjunctivitis, *nAnRC* not asthma and not rhino-connjunctivitis

# according to Allergy diagnostic testing: an updated practice parameter (2008); allergic sensitization was defined as at least one positive skin prick test (SPT)

*according to ATS/ERS guidelines and normalized in accordance with the Global Lungs Initiative 2012

*p*-values come from Pearson’s test for categorical variable or ANOVA test for mean comparison; bold values indicate significance (*p*-values < 0.05)

Chromosome, gene, SNP name, quality control tag, alleles coding (Major/minor), minor allele frequency (MAF), test for Hardy-Weinberg Equilibrium, percentage of missing values (%) and genotyping distribution are reported in Table 2.

**Table 2:** Characteristics of the 52 Single Nucleotide Polymorphisms (SNPs)

Chr	Gene	SNP name	QC	Alleles (M/m)	MAF	HWE *p* values	Missing (%)	GENO (AA/Aa/aa)
1	SELE	rs5361	–	T/G	9.9	0.005	5.4	814/161/18
2	ORMDL1	rs5742940	–	G/A	1.8	0.028	1.0	1004/33/2
3	CACNA2D2	rs12488468	LCR	G/T	48.2	0.014	25.9	187/432/159
3	DOCK3	rs76699816	HWEd	G/A	11.6	**< 0.001**	8.7	779/138/42
4	TLR1	rs17616434	–	T/C	47.1	0.020	5.6	295/459/237
4	TLR1	rs2101521	HWEd/LCR	G/A	34.4	**< 0.001**	15.6	444/274/168
4	TLR1	rs4833095	HWEd	T/C	48.4	**< 0.001**	7.1	292/422/261
4	TLR1	rs5743595	HWEd	A/G	30.5	**< 0.001**	5.8	506/363/120
4	TLR10	rs10004195	HWEd/LCR	T/A	46.2	**< 0.001**	11.0	309/387/238
4	TLR10	rs4274855	LCR	C/T	29.4	1.000	22.5	408/334/72
4	TLR2	rs11736691	GF	–	–	–	100.0	–
4	TLR6	rs1039560	–	T/C	33.4	0.126	3.3	455/442/118
4	TLR6	rs5743789	HWEd/LCR	A/T	29.5	**< 0.001**	19.8	460/268/114
5	IL13	rs1800925	–	C/T	18.4	0.721	4.6	668/300/34
5	IL13	rs1881457	–	A/C	18.4	0.720	2.8	681/305/35
5	IL13	rs20541	HWEd	G/A	14.5	**< 0.001**	7.1	734/200/41
6	IL17	rs7741835	–	C/T	19.4	0.371	5.0	659/291/48
9	DMRT1	rs3812523	–	A/G	15.6	0.074	3.4	733/245/36
9	IL33	rs1342326	–	A/C	21.8	0.174	3.5	627/331/55
9	IL33	rs928413	–	A/G	30.8	0.397	4.3	486/418/101
11	ANO9	rs7482596	HWEd	G/T	13.3	**< 0.001**	4.2	770/205/31
11	ANO9	rs7484182	HWEd	T/C	14.1	**< 0.001**	4.1	758/215/34
11	DHCR7	rs1044482	GF	–	–	–	100.0	–
11	GST-P1	rs1695	HWEd	A/G	29.9	**< 0.001**	6.2	521/338/126
11	NADSYN1	rs2186777	–	A/C	26.7	0.014	4.5	553/365/85
11	SIGIRR	rs4074794	HWEd	G/A	19.5	**< 0.001**	6.3	659/266/59
12	IRAK3	rs1152918	–	C/T	6.9	0.295	2.1	893/128/7
12	IRAK3	rs2701652	–	G/C	22.0	0.256	4.1	620/330/57
12	ORMDL2	rs7954619	GF	–	–	–	100.0	–
16	IL4R	rs1801275	–	A/G	16.0	0.001	4.7	722/237/42
16	IL4R	rs1805012	HWEd	T/C	5.8	**< 0.001**	2.6	916/96/11
16	IL4R	rs3024548	HWEd/LCR	C/G	46.4	**< 0.001**	13.0	314/351/249
17	ERBB2	rs1058808	–	G/C	30.6	0.009	6.8	495/369/115
17	ERBB2	rs1136201	HWEd	A/G	13.8	**< 0.001**	4.1	767/203/37
17	ERBB2	rs2934971	GF	–	–	–	100.0	–
17	ERBB2	rs2952155	GF	–	–	–	100.0	–
17	ERBB2	rs4252665	–	C/T	1.7	0.137	1.6	999/33/1
17	GSDMA	rs3859192	–	C/T	37.9	0.028	6.5	398/423/161
17	GSDMA	rs3894194	–	G/A	41.6	0.005	5.3	359/442/193
17	GSDMA	rs7212938	–	T/G	44.2	0.039	4.8	326/463/211
17	GSDMB	rs2305479	–	C/T	42.0	0.166	1.9	355/485/190
17	GSDMB	rs2305480	–	G/A	40.2	0.088	3.8	371/466/173
17	GSDMB	rs7216389	HWEd	T/C	42.2	**< 0.001**	6.6	359/416/206
17	LRRC3C	rs8065126	HWEd/LCR	C/T	38.3	**< 0.001**	12.5	386/362/171
17	LRRC3C	rs8079416	–	T/C	45.2	0.013	4.5	315/469/219
17	MAP2K3	rs10468608	HWEd/LCR	C/T	30.2	**< 0.001**	19.8	462/251/129
17	MAP2K3	rs2363226	GF	–	–	–	100.0	–
17	MAP2K4	rs3760201	HWEd/LCR	A/G	32.9	**< 0.001**	34.6	343/236/108
17	ORMDL3	rs8076131	HWEd/LCR	A/G	40.7	**< 0.001**	15.9	333/382/168
17	PGAP3	rs1495102	HWEd/LCR	C/T	14.0	**< 0.001**	17.7	697/92/75
17	ZPBP2	rs11557467	GF	–	–	–	100.0	–
X	IRAK1	rs1059703	HWEd	A/G	25.5	**< 0.001**	4.2	679/140/187

*Chr* chromosome, *MAF* minor allele frequency, *GF* genotyping failing, *LCR* low call rate, *HWEd* deviation from the Hardy-Weinberg equilibrium; A: major allele; a: minor allele; bold values indicate significance (*p*-values < 0.001)

By applying the case/control model, no SNP reached the Bonferroni corrected significance threshold (*P* value < 0.002, i.e., 0.05/#tests), and only one SNP reached the Bonferroni corrected suggested significance threshold (*P* value < 0.005, i.e., 0.10/#tests). However, we also included those SNPs reaching the nominal significance threshold (*P* value < 0.05) just to highlight modest associations with the two studied phenotypes. CA was strongly associated only with rs4252665 and modestly with rs1801275 and rs17616434; RC was modestly associated with rs7741835, rs8079416, rs3859192, rs3894194 and rs7212938. Table 3 shows the genotypic frequencies of the associated SNPs in CA/RC and nAnRC groups, and the adjusted OR and 95% CI from the logistic regression model for the only SNP strongly associated. The SNP rs4252665 showed significantly different genotypic frequencies between the two groups, i.e., the CA subjects had a high frequency of CT heterozygote genotype compared with nAnRC, which were mostly homozygous. Indeed, using the overdominant genetic model, in which the baseline is the homozygous genotype (CC/TT), the CT genotype of rs4252665 showed a large increased risk of CA (OR_C/*T*_ = 5.75; 95% CI = 2.03–16.29). The SNPs modestly associated with CA showed a high frequency of the major allele homozygote genotype compared with nAnRC, in which the genotypes were mostly characterized by the presence of the minor allele. Furthermore, with respect to nAnRC, SNPs modestly associated with RC showed small variations in the genotypic frequencies. In particular, the SNPs rs8079416, rs7741835 and rs3894194 showed high frequencies of heterozygote genotypes compared with nAnRC, which are frequently homozygous, whilst the SNPs rs7212938 and rs3859192 had high frequencies of the minor allele homozygote genotypes compared with nAnRC, in which genotypes are characterized by the presence of the major allele.

**Table 3:** Genotypic frequencies of the associated SNPs in CA/RC and nAnRC groups

Gene	Region	SNP name	Alleles	Group	AA (%)	Aa (%)	aa (%)	OR (95% CI)
TLR1	4p14	rs17616434	T/C	nAnRC	29.4	45.6	25.0	–
				CA	42.4	44.1	13.5	
IL4R	16p12.1	rs1801275	A/G	nAnRC	71.0	24.5	4.5	–
				CA	80.0	20.0	0.0	
ERBB2	17q12	rs4252665	C/T	nAnRC	97.4	2.5	0.1	5.75 (2.03–16.29)
				CA	90.2	9.8	0.0	
IL17	6p12.2	rs7741835	C/T	nAnRC	64.3	31.2	4.5	–
				RC	69.1	25.5	5.4	
LRRC3C	17q21.1	rs8079416	T/C	nAnRC	32.8	45.0	22.2	–
				RC	33.9	42.4	23.7	
GSDMA	17q21.1	rs7212938	T/G	nAnRC	34.1	45.4	20.5	–
				RC	32.8	41.4	25.8	
GSDMA	17q21.1	rs3859192	C/T	nAnRC	42.2	42.6	15.2	–
				RC	40.7	40.7	18.6	
GSDMA	17q21.1	rs3894194	G/A	nAnRC	37.9	43.3	18.8	–
				RC	39.0	37.3	23.7	

A: major allele; a: minor allele

Adjusted odds ratios (OR) and 95% confidence interval (CI) of the logistic regression models

The detected association signals for CA were located within the Toll-like receptor (TLR1) on chromosome 4, the interleukin 4 receptor (IL4R) on chromosome 16 and the Erb-b2 receptor tyrosine kinase 2 (ERBB2) on chromosome 17. It is known that Toll-like receptors (TLRs) represent a major group of receptors for the specific recognition of pathogen-associated molecular patterns of microbes capable of activating innate and adaptive immunity that reduce the risk for asthma [3]. The IL4R gene is known to encode a protein that regulates IgE production and it has been shown that allelic variations in this gene are associated with atopy, allergic rhinitis and asthma [4]. Recently, some loci of ERBB2, which belong to the encoding region 17q12, have been reported to be in linkage disequilibrium with loci in the region 17q21 encoding gasdermin A (GSDMA) gene, previously associated with childhood asthma [5–7].

With regard to the RC, the modestly associated genes were interleukin 17 (IL17) on chromosome 6, leucine rich repeat containing 3C (LRRC3C), and GSDMA on chromosome 17. IL17 is a pro-inflammatory cytokine that targets epithelial cells48 and its expression in the nasal mucosa has been associated with allergic rhinitis and its degree of severity [8, 9]. To our knowledge, no functional studies have been published on LRRC3C, although, within the human genome, the gene LRRC32 has been associated with eczema and allergic rhinitis [10], and probably some similarities between the two proteins encoded by LRRC3C and LRRC32 exist. Finally, GSDMA gene has been associated with childhood asthma and allergic disease in many populations. In particular, region 17q21 has been originally identified in the first GWAS on childhood asthma [6], and GSDMA variants were suggested to be strong risk factors for asthma and airway inflammation [7].

Overall, our findings reveal the importance of different genetic variants at 4p14, 16p12.1 17q12, 6p12.2 and 17q21.1, identifying possible candidate genes responsible for CA and RC in the Sicilian child population. These results are a preliminary step in understanding the pathophysiology of asthma and rhino-conjunctivitis in a paediatric population in the Mediterranean area and need to be verified by further studies using more advanced technologies. Furthermore, novel methodologies combining genome-wide association study (GWAS; [11]) and expression quantitative trait locus (eQTL [12]) such as summary-data based Mendelian randomization (SMR; [13]), PrediXcan [14], MetaXcan [15], would be useful in discovering new genetic variants linked to these allergic respiratory diseases in this geographic area. Unlike traditional single-variant tests, these innovative approaches based on SNP-gene linkage will provide valuable insights on disease causality. Noteworthy, the integrative analysis of GWAS and eQTL studies, by identifying gene-trait-associated changes in the expression, would mitigate some tasks associated with a GWAS approach, allowing the discover of genetic variants which can affect gene expression [16]. Moreover, since some gene functions are often pleiotropic, this combined approach would allow a better comprehension of the pathways through which pleiotropy can affect clinical phenotypes.

In conclusion, the present study could facilitate the application of novel therapeutics and preventive strategies arising from the genomics era of precision medicine.

## Abbreviations

BMI: Body mass index
CA: Current Asthma
eQTL: Expression quantitative trait locus
ERBB2: Human epidermal growth factor receptor 2
GSDMA: Gasdermin A
GWAS: Genome-wide association study
HWE: Hardy-Weinberg equilibrium
IL17: Interleukin 17
IL4R: Interleukin 4 receptor
LRRC3C: Leucine rich repeat containing 3C
MAF: Minor allele frequency
nAnRC: Not asthma and not rhino-conjunctivitis
RC: Rhino-conjunctivitis
SMR: Summary-data based Mendelian randomization
SNP: Single Nucleotide Polymorphism
SPT: Skin prick tests
TLR: Toll-like receptor
